# Sex and strain differences in renal hemodynamics in mice

**DOI:** 10.14814/phy2.15644

**Published:** 2023-03-22

**Authors:** Yu Tao, Cassandra Young‐Stubbs, Parisa Yazdizadeh Shotorbani, Dong‐Ming Su, Keisa W. Mathis, Rong Ma

**Affiliations:** ^1^ Department of Physiology and Anatomy University of North Texas Health Science Center Fort Worth Texas USA; ^2^ Department of Microbiology, Immunology and Genetics University of North Texas Health Science Center Fort Worth Texas USA

**Keywords:** glomerular filtration rate, renal blood flow, renal hemodynamics, sex, strain

## Abstract

The present study was to examine sex and strain differences in glomerular filtration rate (GFR) and renal blood flow (RBF) in C57BL6, 129/Sv, and C57BLKS/J mice, three commonly used mouse strains in renal research. GFR was measured by transdermal measurement of FITC‐sinitrin clearance in conscious mice. RBF was measured by a flow probe placed in the renal artery under an anesthetic state. In C57BL6 mice, there were no sex differences in both GFR and RBF. In 129/Sv mice, females had significantly greater GFR than males at age of 24 weeks, but not at 8 weeks. However, males had higher RBF and lower renal vascular resistance (RVR). Similar to 129/Sv, female C57BLKS/J had significantly greater GFR at both 8 and 24 weeks, lower RBF, and higher RVR than males. Across strains, male 129/Sv had lower GFR and higher RBF than male C57BL6, but no significant difference in GFR and greater RBF than male C57BLKS/J. No significant difference in GFR or RBF was observed between C57BL6 and C57BLKS/J mice. Deletion of eNOS in C57BLKS/J mice reduced GFR in both sexes, but decreased RBF in males. Furthermore, there were no sex differences in the severity of renal injury in eNOS^−/−^ *dbdb* mice. Taken together, our study suggests that sex differences in renal hemodynamics in mice are strain and age dependent. eNOS was not involved in the sex differences in GFR, but in RBF. Furthermore, the sexual dimorphism did not impact the severity of renal injury in diabetic nephropathy.

## Graphical abstract

There are sex and strain differences in renal hemodynamics, which may also be age dependent. The sex difference in renal hemodynamics is not attributed to eNOS, and do not impact the development of diabetic kidney disease.

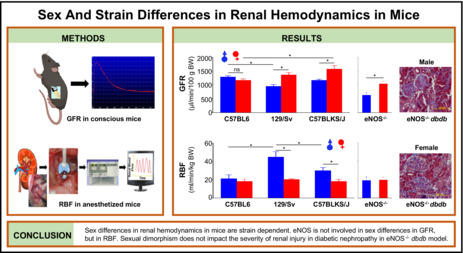

## 1 INTRODUCTION

It is increasingly recognized that interactions between biological, social, and environmental factors play a significant role in the prevention, pathophysiology, and management of human disease (Mannon et al., 2020). Sex/gender differences and race/ethnicity/genetic background are among those factors with a significant impact on human health and disease. It is evident that there are sex differences in the incidence, age of onset, manifestation, severity and development, as well as response to treatment in various diseases, including heart disease, hypertension, obesity, and acute or chronic renal ischemia (Arnold et al., 2017; Gillis & Sullivan, 2016; Kher et al., 2005; Swartling et al., 2022). In kidneys, sex differences have been reported in gene expressions, transporters, mitochondrial function, circadian clock, structure and/or function, and in the occurrence and development of several kidney diseases, such as diabetic nephropathy (DN) (Clotet et al., 2016; Harris et al., 2018; Institute for Health Metrics and Evaluation, 2020; Layton & Gumz, 2022; Maric‐Bilkan, 2020; Skrtic et al., 2017; Sultanova et al., 2020; Veriras et al., 2017). In addition to sex differences, racial heterogeneity in renal function (Hsu et al., 2021), and in the incidence and prevalence of kidney diseases (GBD, 2018; Jha et al., 2013; Power, 2019; Susantitaphong et al., 2013) is evident. Animal studies demonstrated that genetic factors play a major role in determining susceptibility, as well as resistance, to kidney disease. For instance, previous studies identified significant strain differences affecting the development of diabetic renal complications in mice (Breyer et al., 2005; Brosius et al., 2009; Gurley et al., 2005, 2009).

The mouse is a widely used tool for studying renal function and developing models of human kidney diseases because its genome is tractable for manipulation and its diverse and unique genetic resources are accessible (Beck et al., 2000; Bronson & Smithies, 1994; Marshall et al., 1992). In the present study, we studied the sex and strain differences in glomerular filtration rate (GFR) and renal blood flow (RBF) in three strains of mice which are commonly‐used in renal research. We further explored the role of endothelial nitric oxide synthase (eNOS) in the differences and the impact of the differences on the development of DN. The rationales of the study included (1) there are very limited data to compare sex and strain differences in renal hemodynamics in mice, (2) there are lack of studies on association of the differences in renal hemodynamics with severity of kidney disease, such as DN, and (3) there are very limited study to measure GFR in mice using a non‐invasive approach under conscious and free‐moving state. The information from this study would help researchers in the field appropriately design experiments and evaluate data when using a mouse model for studying renal physiology and pathology.

## 2 MATERIALS AND METHODS

### 2.1 Animal preparation

Three strains with five genetic backgrounds of mice were included in this study. Except for C57BLKS/J mice, which were purchased from the Jackson Laboratory (Stock #: 000662), all other strains of mice were inbred mice and maintained at animal facility of University of North Texas Health Science Center at Fort Worth. The C57BL/6 breeders were purchased from Charles River Laboratory. The 129/Sv mouse breeders were obtained from Dr. Mary B. Humphrey at University of Oklahoma Health Sciences Center. eNOS^−/−^ and eNOS^−/−^ *db/db* mice were generated from homozygous eNOS^−/−^ and heterozygous Lepr^db^ (eNOS^−/−^ *db*) on C57BLKS/J background. The eNOS^−/−^ *db* breeding pairs were purchased from The Jackson Laboratory (JAX# 8340). The eNOS^−/−^ and eNOS^−/−^ *db/db* mice were identified by genotyping at an age of 3–4 weeks using high‐resolution melting PCR protocol provided by the vendor and publications in (Mohan et al., 2008; Shesely et al., 1996).

The age of both male and female mice used in this study ranged from 8 to 24 weeks. During renal blood flow measurements, mice were placed in an induction chamber containing 4% isoflurane anesthesia and thereafter maintained on isoflurane (2% in oxygen, delivered through a mask) for the duration of surgery. All procedures were approved by the University of North Texas Health Science Center Institutional Animal Care and Use Committee. All mice were maintained at the animal facility of University of North Texas Health Science Center under local and NIH guidelines. These mice were housed in a specific pathogen‐free facility with a temperature‐controlled room, and regulated with 12‐h light/dark cycle and free access to water and food (standard chow diet: LabDiet) containing 25.1% fiber, 0.29% Na^+^, 19.3% protein, 13.5% fat, and 15.8% calories from fat.

### 2.2 Blood pressure measurements

A catheter was inserted into the left carotid artery of mice under isoflurane anesthesia. Blood pressure was continuously recorded during the entire period of RBF measurement in anesthetized mice using PowerLab software as previously reported (Chaudhari et al., 2022; Fairley & Mathis, 2017). The mean arterial pressure (MAP) averaged from 30‐min continuous recordings after completion of surgery and stabilization of blood pressure was presented in this study.

### 2.3 Assessment of RBF and vascular resistance

Following a posterior incision, the right renal artery was isolated from the corresponding vein in a subset of anesthetized mice and placed in a Transonics flow probe (Model #: 0.5 PS) in order to measure RBF. RBF (mL/min/kg body weight) and anesthetized MAP (mm Hg) were gathered simultaneously for 30 min following a 30‐min stabilization period using Powerlab software. Renal vascular resistance (RVR; mm Hg/min/kg body weight) was calculated by dividing anesthetized MAP by normalized RBF.

### 2.4 Transdermal measurement of GFR

GFR was measured in conscious, freely moving mice using transdermal measurement of sinistrin clearance rate as previously reported (Chaudhari et al., 2022). Anesthetized mice were positioned on a surgery plate and the right dorsal hair was shaved with an electrical shaver followed by the application of depilatory lotion and 70% ethanol to remove any residual hair. The transdermal fluorescence detector (MB 0309 Mini, MediBeacon Inc.) was directly attached to the naked skin and fixed to the mouse body using medical tape. FITC‐conjugated sinistrin was then administered by retro‐orbital injection at 0.03 mg/g body weight (0.03–0.05 mL) using a 0.5 mL BD insulin syringe (28G × ½”). The mouse was recovered from isoflurane about 10–20 s. The excitation kinetics of the exogenous GFR tracer were recorded using the software provided by the vendor (MB Lab Ver. 2.18) in freely‐moving, conscious mice for 1.5 h. The recorded sinistrin clearance curve was fitted in the software provided by the vendor (MB Studio Ver. 2.1) using a two‐compartment model. GFR was calculated based on the half‐life (t_1/2_) of plasma FITC‐sinistrin decay using the formula 14616.8/(t1/2) and reported as μL/min/100 g of body weight as previously described (Scarfe et al., 2018; Schock‐Kusch et al., 2013; Schreiber et al., 2012).

### 2.5 Assessment of urinary albumin excretion rate

Urine samples were collected through metabolic cages (Catalog #: 370 0 M022, Braintree Scientific Inc.) during the period of 24 h. Urinary albumin and creatinine levels were determined using Albuwell‐M kits (Exocell). Albumin excretion rate was expressed as the ratio of urinary albumin concentration to urinary creatinine concentration (ACR, μg/mg) (Ma, Li, et al., 2019).

### 2.6 Renal tissue preparation and immunohistochemical staining

Mice were anesthetized by intraperitoneal injection of ketamine with xylazine (100 + 10 mg/kg). The mice were perfused with physiological saline solution through the left ventricle to wash out blood, followed by perfusion with 4% paraformaldehyde. The right kidneys were excised, decapsulated, cut in half through a midsagittal plane, and fixed with 4% paraformaldehyde. The fixed kidneys were dehydrated through a graded series of ethanol, infiltrated and embedded in paraffin, sectioned (4–5 μm), and mounted on glass slides. For immunohistochemical staining, kidney sections were deparaffinized. Antigen retrieval was achieved by heating the sections in 10 mM citrate buffer in a microwave for 10 min. The sections were blocked by 5% goat serum for 30 min at room temperature and then incubated with anti‐Wilms' Tumor 1 (WT1) antibody (rabbit polyclonal, catalog #: MBS9203569, lot #: SA170311DC, MyBioSource) at 1:20 at 4°C overnight. Secondary antibody was Alexa 568‐conjugated antibody (goat anti‐rabbit) from Invitrogen. Nuclei were stained in blue using DAPI. Slides were coverslipped using resinous mounting medium. Sections were examined using an Olympus microscope (BX41) and an Olympus DP70 digital camera with DP manager software (version 2.2.1). Images were uniformly adjusted for brightness and contrast. Counting of WT1 positive cells was conducted by a blinded observer using ImageJ (version 1.50b; NIH). The representative photomicrographs in Figure 8c were taken with a Zeiss inverted confocal microscope (Zeiss LSM 880).

### 2.7 Statistical analysis

**FIGURE 1:**
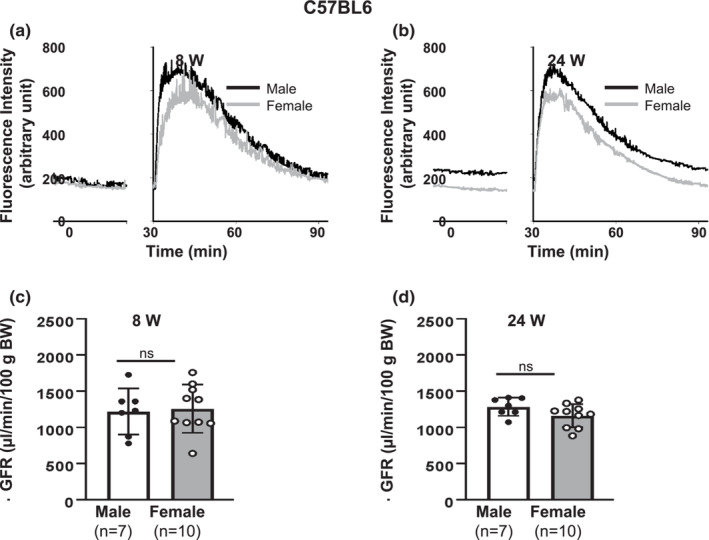
GFR in male and female conscious C57BL6 mice. (a, b) Representative FITC‐sinistrin clearance curves, showing plasma fluorescence signals measured transcutaneously after FITC‐sinistrin application in both male and female C57BL6 mice at 8 weeks (8 W, a) and 24 weeks (24 W, b). (c, d) Summary of calculated GFR from groups presented in a and b, respectively. ns: no significant difference, males versus. females (Unpaired Student's *t*‐test). “*n*”: the number of mice; ns, no significant difference, males versus females (Unpaired Student's *t*‐test).

**FIGURE 2:**
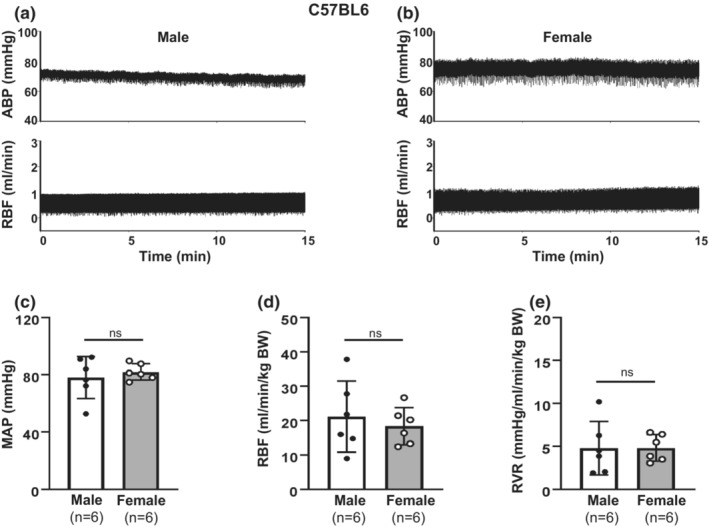
Comparison of renal hemodynamics between male and female C57BL6 mice. (a, b) Representative arterial blood pressure (ABP) and RBF recordings in a male (a) and a female (b) mouse at age of 24 weeks. (c) Summary data of anesthetized mean arterial blood pressure (MAP) from six male and six female mice (*n* = 6). (d) Summary data of RBF from seven male and seven female mice (*n* = 7). RBF was normalized to BW. (e) Summary data of calculated renal vascular resistance (RVR) from six male and six female mice (*n* = 6). RVR was normalized to BW. From (c–e), “ns” indicates no significant difference (Unpaired Student's *t*‐test).

**FIGURE 3:**
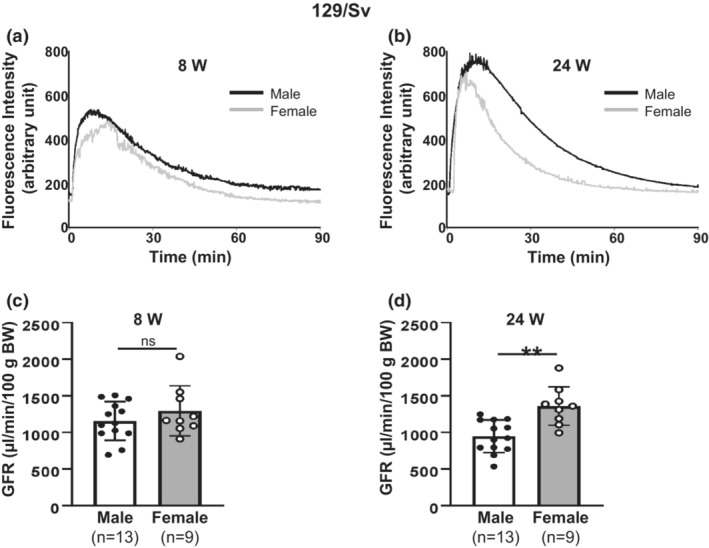
Sex difference in GFR in conscious 129/Sv mice. (a, b) Representative FITC‐sinistrin clearance curves, showing plasma fluorescence signals after FITC‐sinistrin application in both male and female 129/Sv mice at 8 weeks (8 W, a) and 24 weeks (24 W, b). (c, d) Summary of calculated GFR from groups presented in (a, b), respectively. ns: no significant difference. “**” denotes *p* < 0.01, males versus females (Unpaired Student's *t*‐test). “*n*”: the number of mice.

**FIGURE 4:**
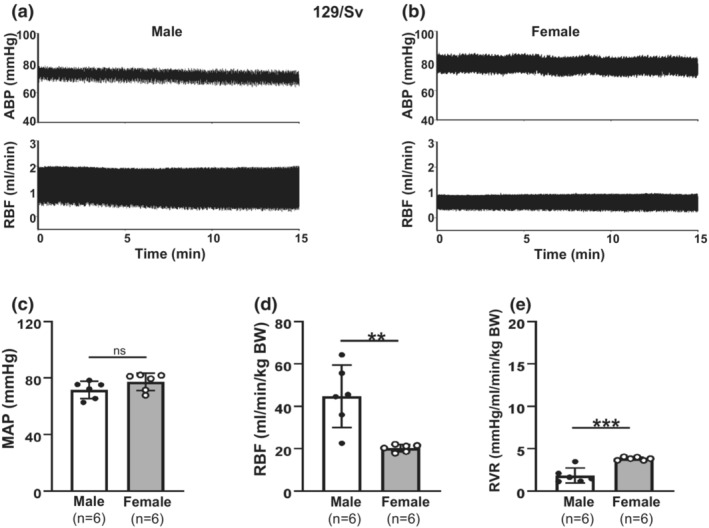
Sex difference in renal hemodynamics in 129/Sv mice. (a, b) Representative ABP and RBF recordings in a male (a) and a female (b) mouse at age of 24 weeks. (c) Average anesthetized MAP from seven male and six female mice. (d) Summary data of RBF from seven male and six female mice. RBF was normalized to BW. (e) Summary data of calculated RVR from seven male and six female mice. RVR was normalized to BW. From (c–e), “ns” indicates no significant difference, ** denotes *p* < 0.01, and *** denotes *p* < 0.001 (Unpaired Student's *t*‐test).

**FIGURE 5:**
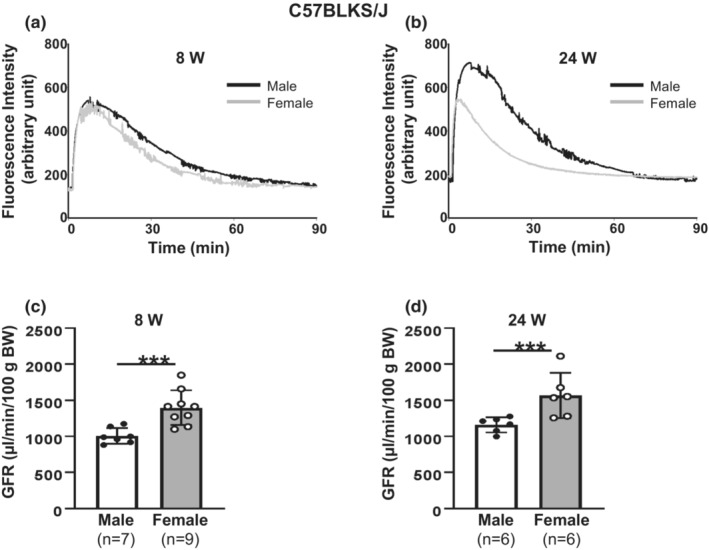
Sex differences in GFR in conscious C57BLKS/J mice. (a, b) Representative FITC‐sinistrin clearance curves, showing plasma fluorescence signals after FITC‐sinistrin application in both male and female C57BLKS/J mice at age of 8 weeks (8 W, a) and 24 weeks (24 W, b). (c, d) Summary of calculated GFR from groups presented in (a, b), respectively. GFR was normalized to BW. “***” denotes *p* < 0.01, males versus females (Unpaired Student's *t*‐test). “*n*”, the number of mice.

**FIGURE 6:**
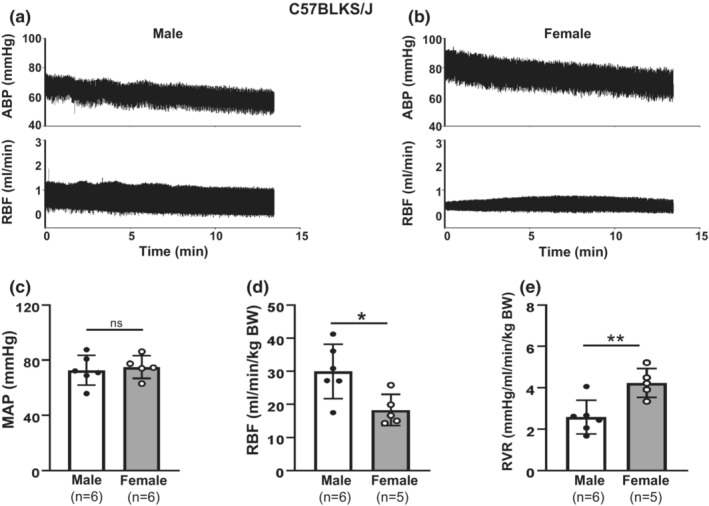
Sex differences in renal hemodynamics in C57BLKS/J mice. (**a, b)** Representative ABP (upper panels) and RBF (bottom panels) recordings in a male (a) and a female (b) mouse at age of 22–24 weeks. (**c)** Average anesthetized MAP from six male and five female mice. (**d)** Summary data of RBF from six male and five female mice. RBF was normalized to BW. (e**)** Summary data of calculated RVR from six male and five female mice. RVR was normalized to BW. From (c–e), “ns” indicates no significant difference, * denotes *p* < 0.05, and ** denotes *p* < 0.001 (Unpaired Student's *t*‐test).

**FIGURE 7:**
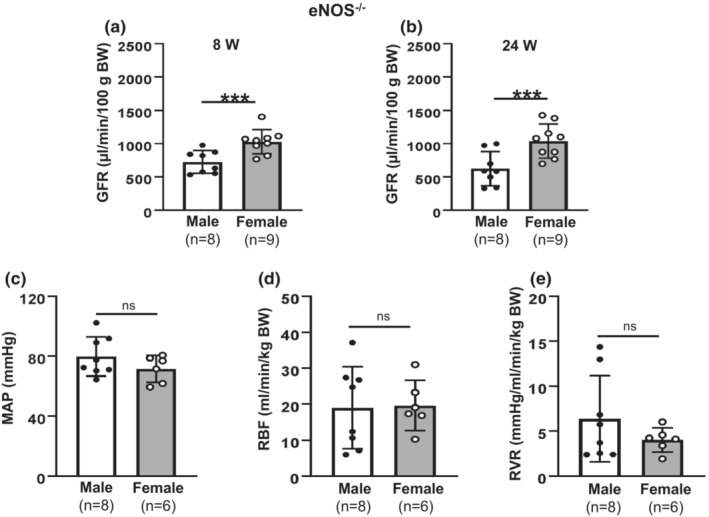
GFR and renal hemodynamics in eNOS‐knocked out male and female C57BLKS/J mice. (a, b) GFR in both male and female eNOS^−/−^ C57BLKS/J mice at ages of 8 weeks (8W) (a) and of 24 weeks (24 W) (b). “***” denotes *p* < 0.01, males versus females (Unpaired Student's *t*‐test). “*n*”: the numbers of mice. (c) Anesthetized MAP from eight male and six female eNOS^−/−^ mice. (d) Summary data of RBF from eight male and six female mice. (e) Summary data of calculated RVR from eight male and six female mice. From (b–d), the age of mice was 24 weeks. “ns” indicates no significant difference (Unpaired Student's *t*‐test).

**FIGURE 8:**
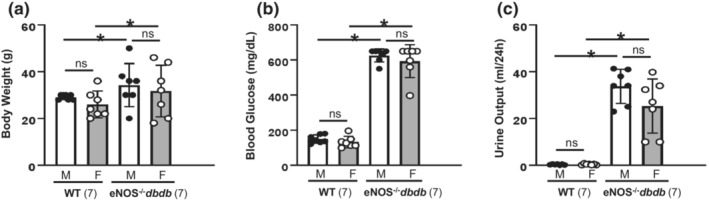
Body weight (a), blood glucose level (b), and urine output (c) of male (M) and female (F) wild‐type C57BLKS/J (WT, nondiabetic control) and eNOS^−/−^ *db/db* mice. “ns” indicates no significant difference; “*” denotes *p* < 0.05 (One‐way ANOVA), compared to the groups as indicated. The mice in all groups were at age of 20 weeks. The number in parenthesis represents the number of mice each group.

**FIGURE 9:**
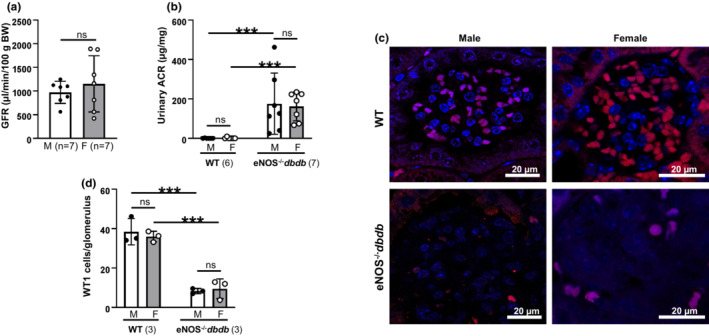
Development of renal injury in male and female eNOS^−/−^ *dbdb* mice. (a) GFR in male (M) and female (F) eNOS^−/−^ *dbdb* mice at age of 20 weeks. “ns” denotes no significant difference (Unpaired Student's *t*‐test). (b) Albumin excretion rate of male and female WT and eNOS^−/^−*dbdb* mice at age of 20 weeks. Albumin excretion rate is indicated by urinary albumin creatinine ratio (ACR). “ns” indicates no difference, “***” denotes *p* < 0.001 (One‐way ANOVA), comparisons between groups as indicated. The number in parenthesis indicates the number of mice each group. (c) Representative high‐magnification images of podocyte staining with Wilms' tumor 1 (WT1, red) in a male and a female WT and eNOS^−/−^ *dbdb* mouse at 20 weeks. Nuclei were counterstained with DAPI (blue). (d) Number of podocytes (WT1‐positively stained cells) per glomerulus averaged from three male and female WT and eNOS−/−dbdb mice. About 15–20 glomeruli from one kidney section were counted and five sections were taken from one mouse. “ns” indicates no difference, “***” denotes *p* < 0.001 (One‐way ANOVA), comparisons between groups as indicated.

Data were reported as means ± SD. Student's unpaired *t*‐test was used to identify the difference between two groups (Figures 1, 2, 3, 4, 5, 6, 7 and 9a). The one‐way ANOVA plus Student–Newman–Keuls post hoc analysis was used to analyze the differences in one parameter among multiple groups (Figures 8 and 9b,c). The two‐way ANOVA plus Tukey's honestly significant difference post hoc analysis was used to analyze the differences in two parameters among multiple groups (Tables 1 and 2). *p* < 0.05 was considered statistically significant. Statistical analyses were performed using SigmaStat (Jandel Scientific).

**TABLE 1:** Body weight (g) of male and female mice with different strains or genotypes.

Mouse strain/Genotype	8 weeks		24 weeks	
	Male	Female	Male	Female
C57BL6	26.6 ± 1.0 (7)	20.4 ± 1.3 (10)^b^	34.6 ± 3.2 (7)^c^	32.4 ± 4.3 (10)^e^
129/Sv	22.6 ± 2.6 (13)	20.2 ± 3.2 (9)	28.3 ± 3.1 (13)^c^	24.9 ± 3.3 (9)^a^
C57BLKS/J	24.0 ± 1.2 (7)	20.0 ± 1.4 (9)^b^	28.9 ± 1.2 (7)^c^	24.2 ± 1.9 (9)^b^, ^d^
eNOS^‐/‐^	26.4 ± 1.1 (9)	21.8 ± 1.0 (9)^b^	33.9 ± 3.1 (9)^c^	27.2 ± 2.9 (9)^b^, ^d^

*Note*: Data are expressed as mean ± SD.

^a^ Denotes *p* < 0.05.

^b^ Denotes *p* < 0.001, comparison between male and female mice at the same age and strain or genotype.

^c^ Denotes *p* < 0.05.

^d^ Denotes *p* < 0.01.

^e^ Denotes *p* < 0.001, comparison between 8 and 24 weeks of the same sex and strain or genotype of mice. The number inside parenthesis indicates the number of mice in each group.

**TABLE 2:** Strain differences in GFR and RBF in male and female mice.

Mouse strain	GFR (μL/min/100 g BW)		RBF (mL/min/kg BW)	
	Male	Female	Male	Female
C57BL/6	1284.5 ± 123.9 (7)	1162.9 ± 159.4 (10)	20.3 ± 9.7 (7)	17.6 ± 5.4 (7)
129/Sv	946.5 ± 222.5 (13)^a^	1359.8 ± 263.8 (9)	44.8 ± 18.5 (6)^a^, ^b^	20.3 ± 1.7 (6)
C57BLKS/J	1161.3 ± 105.3 (6)^c^	1568.3 ± 312.4 (6)^a^, ^c^	30.0 ± 8.2 (6)^c^	18.3 ± 4.7 (5)
eNOS^‐/‐^	626.3 ± 189.3 (8)	1039.7 ± 144.9 (9)^a^	19.0 ± 11.5 (8)	19.6 ± 6.9 (6)

*Note*: Data are expressed as mean ± SD.

^a^ Denotes *p* < 0.05, compared with the same sex of C57BL6 mice.

^b^ Denotes *p* < 0.05, compared with the same sex of C57BLKS/J mice.

^c^ Denotes *p* < 0.05, compared with eNOS^‐/‐^ mice with the same sex. All mice were at the age of 22—24 weeks. The number inside parenthesis indicates the number of mice in each group.

## 3 RESULTS

### 3.1 GFR in male and female C57BL6 mice

C57BL6 mouse is a multipurpose model that is the most widely used for research into physiology and pathophysiology. In the present study, young adult (8 weeks old) and adult (24 weeks old) C57BL6 mice were used. Body weight (BW) of male mice was significantly greater than that of females at 8 weeks. At 24 weeks, BW from both male and female mice were significantly increased compared to themselves at 8 weeks. However, there was no significant sex difference at this age (Table 1).

Transcutaneous measurement of GFR was conducted in conscious C57BL6 mice at both 8 weeks and 24 weeks. As shown in Figure 1a,b, the profiles of plasma FITC‐sinistrin decay were very similar in both male and female mice at both ages. The normalized GFR calculated from the sinistrin clearance rate was not significantly different between males and females either at 8 or 24 weeks (Figure 1c,d), suggesting there was no sex difference in GFR in conscious C57BL6 mice.

### 3.2 RBF in male and female C57BL6 mice

MAP and RBF were simultaneously measured in male and female C57BL6 mice under anesthesia at an age of 22–24 weeks. Representative tracings of raw MAP and RBF are shown in Figure 2 a (male) and b (female). The summary data are presented in Figure 2c,d. There were no significant sex differences in MAP, RBF (Figure 2c,d) or calculated RVR (Figure 2e) observed.

In summary, the results in Figures 1 and 2 suggest that there were no significant sex differences in renal hemodynamics in C57BL6 mice.

### 3.3 Sex differences in GFR in conscious 129/Sv mice

129/Sv mouse is a substrain of 129 mouse which is one of most commonly used strains of mice for renal research (Lu et al., 2012) and is an important strain of mice for creating “knockout” and other targeted mutant mice (Simpson et al., 1997). To explore sex differences in GFR in this strain, we measured GFR in conscious males and females at age of 8 weeks and 24 weeks. No sex differences in BW were observed at 8 weeks. However, at 24 weeks, the BW of female mice was significantly lower than that of male mice (Table 1). The rate of plasma sinistrin decay was similar between male and female mice at 8 weeks, but much faster in female mice at 24 weeks (Figure 3a,b). Consistently, the calculated GFR revealed no sex difference at 8 weeks, but significantly greater in female mice compared to the males at 24 weeks (Figure 3c,d).

### 3.4 Sex differences in RBF in 129/Sv mice

Sex differences in renal hemodynamics have not been studied in 129/Sv mice previously. We compared MAP and RBF between adult male and female 129/Sv mice under anesthetic states. As shown in Figure 4, no significant difference in MAP between males and females was observed. However, there were significant sex differences in RBF with males significantly greater than females. Thus, the calculated RVR in male mice was significantly lower than that in female mice.

### 3.5 Sex differences in GFR in conscious C57BLKS/J mice

Although C57BLKS/J is closely related to C57BL/6J, the two strains are phenotypically distinct. The mutations diabetes (*Lepr* ^*db*^) and obese (*Lep* ^*ob*^) each express a much more severe phenotype on the C57BLKS/J background than on the C57BL6 background. Since this strain is more susceptible to renal disease than C57BL6, C57BLKS/J mouse has been used to develop a widely‐accepted model of DN (Leiter et al., 1999; Norgaard et al., 2019; Stec et al., 2015; Xue et al., 2019; Zhang et al., 2012; Zhao et al., 2006).

Sex differences in GFR in C57BLKS/J mice have not been studied previously. We measured GFR in young adult (8 weeks) and adult (24 weeks) male and female C57BLKS/J mice in the present study. Similar to C57BL6, but different from 129/Sv mice, the BW of female C57BLKS/J mice was significantly lower than that of males at 8 weeks. Also, the female C57BLKS/J at 24 weeks was significantly lighter than males, the same as in 129/Sv mice, but different from C57BL6 mice. Furthermore, like C57BL6 mice, both male and female C57BLKS/J mice at 24 weeks were significantly heavier than those of the same sex at 8 weeks (Table 1). The rate of plasma sinistrin clearance was much faster in female mice at both 8 and 24 weeks (Figure 5a,b). Accordingly, the calculated GFR, which was normalized to BW was significantly greater in female mice compared to males at both ages (Figure 5c,d).

### 3.6 Sex differences in RBF in C57BLKS/J mice

We further examined RBF in male and female C57BLKS/J mice under anesthetized state at age of 22–24 weeks. There was no sex difference in MAP (Figure 6ac). However, RBF had significant sex differences. Same as in 129/Sv mice, the normalized RBF of females was significantly lower than that of males (Figure 6a,b,d). The calculated RVR in female mice was significantly greater than that in male mice (Figure 6e).

### 3.7 eNOS did not contribute to sex differences in GFR, but mediated greater RBF in male C57BLKS/J mice

Nitric oxide (NO) is central to the control of vascular homeostasis, blood pressure, and renal function (Layton & Sullivan, 2019). In the kidney, eNOS is localized in vascular endothelial cells, glomerular mesangial cells, and tubules, where it is important in the maintenance of GFR, vascular tone, and RBF (Bachmann & Mundel, 1994; Kone & Baylis, 1997; Schneider et al., 2008; Yuan et al., 2012; Zhang et al., 2007). Published studies demonstrated significant sex differences in NO system with female subjects/animals having greater NOS protein levels (Neugarten et al., 1997), enzymatic activity (Sullivan et al., 2010), NO production (Forte et al., 1998), NO bioavailability (Chen et al., 2017), and a greater capacity for NO to induce vasodilation (Majmudar et al., 2000). If NO derived from endogenous eNOS underlay the sex differences in GFR and RBF, deletion of this enzyme would be expected to remove/minimize the differences.

To explore the role of NO in the sex differences, we further assessed GFR and RBF in male and female eNOS‐knocked out (eNOS^−/−^) C57BLKS/J mice which have shown significant sex differences in GFR and RBF (Figures 5 and 6). Similar to WT C57BL6 mice, BW of female eNOS^−/−^ mice was significantly lower than that of males at both 8 and 24 weeks. And both male and female eNOS^−/−^ mice at 24 weeks were significantly heavier than those of the same sex at 8 weeks (Table 1).

Global deletion of eNOS (eNOS^−/−^) significantly reduced GFR in both males and females (Table 2), but still showed the same pattern of sex differences in GFR as that in WT mice with females significantly greater than males at both 8 and 24 weeks (Figure 7a,b). Anesthetized blood pressure in eNOS^−/−^ mice was comparable with that in WT mice and no sex difference was observed (Figure 7c). However, eNOS knockout eliminated sex differences in RBF and RVR which were observed in WT mice (Figure 7d,e). These results suggest that eNOS played a key role in the sex differences in RBF, but not in GFR.

### 3.8 Comparison of renal injury between male and female C57BLKS/J mice with DN

To study if the sex differences in GFR and RBF led to differences in renal injury, we evaluated and compared renal insufficiency between male and female eNOS^−/−^ *dbdb* mice (on C57BLKS/J genetic background), a well‐known type II diabetes model (Brosius et al., 2009). BW, fasting blood glucose levels, and 24 h urine output of both male and female eNOS^−/−^ *db/db* mice were significantly greater than those of WT (nondiabetic controls) mice with the same age and sex. However, no sex difference in those variables was observed in diabetic mice (Figure 8).

Previous studies from our and other groups (Ma, Li, et al., 2019; Zhao et al., 2006) revealed severe renal injury in this mouse model at week of 20. We measured and compared GFR in both male and female eNOS^−/−^ *dbdb* mice at this age. Although the female WT C57BLKS/J mice (non‐diabetes control) had a significantly greater GFR than males at both early (8 W) and middle ages (24 W) (Figure 5a), there was no sex difference in GFR in mice with severe DN (Figure 9a). These data suggest that sex differences in GFR may not contribute to the renal insufficiency of diabetes in this mouse model of DN.

Consistently, both male and female diabetic mice revealed significantly greater albumin excretion rate at week 20 compared to the sex‐ and age‐matched controls. However, there were no sex differences observed in eNOS^−/−^ *dbdb* mice (Figure 9b). Since podocyte injury and loss is a hallmark of DN (Ma, Chen, et al., 2019; Tao, Chaudhari, et al., 2022; Tao, Mallet, et al., 2022), we furthermore examined the number of podocytes by staining WT1 in kidney sections from male and female diabetic and control mice. As shown in Figure 9c,d, DN significantly reduced the number of podocytes in both sexes of mice. However, there was no significant difference in podocyte loss between male and female eNOS^−/−^ *dbdb* mice. These data suggest that the sex differences in GFR and RBF in C57BLKS/J mice did not result in differences in renal injury in diabetic kidney.

### 3.9 Comparisons of GFR and RBF among different strains of mice

We then compared the basal GFR and RBF at the same sex among C57BL/6, 129/Sv, and C57BLKS/J mice. For male mice, 129/Sv had significantly lower GFR and higher RBF than C57BL/6. Although the GFR of 129/Sv mice had no significant difference from C57BLKS/J mice, the RBF of 129/Sv mice was significantly greater than that of C57BLKS/J mice. There was no significant difference in either GFR and RBF observed between C57BL/6 and C57BLKS/J mice (Table 2).

## 4 DISCUSSION

In the past few years, an explosion of data has emerged concerning sex differences in kidney function and in the development and severity of kidney disease (Dickinson et al., 2013; Layton & Gumz, 2022; Layton & Sullivan, 2019; Maric‐Bilkan, 2020; Sullivan & Gillis, 2017; Veriras et al., 2017). Although sex differences in GFR have been studied in animal models, including mice, there are valid concerns regarding the sensitivity and accuracy of the methods used, and the conditions of animals during experimental procedures. The present study is distinguished from the previous studies in several aspects. First, we used a recently‐developed mini‐fluorescence device which can detect transdermal signal of plasma FITC‐sinistrin (Scarfe et al., 2018; Schock‐Kusch et al., 2013; Schreiber et al., 2012). This non‐invasive assessment of GFR in conscious and freely‐moving mice is superior to that used in previous studies in which GFR was estimated in anesthetized mice or by collecting blood samples at multiple time points (Dickinson et al., 2013; Hackbarth & Hackbarth, 1981; Li et al., 2017; Qi et al., 2004). Second, findings from the present study suggest that sex differences in GFR were strain and age dependent. Among the three inbred strains, C57BL6 mice did not show sex differences in GFR at both young (8 weeks) and adult (24 weeks) ages. However, 129/Sv females had significantly greater GFR than males, but only at 24 weeks. Different from the two strains, both young and adult C57BLKS/J revealed significant sex differences with a greater GFR in females. Furthermore, in contrast to most studies which reported a higher GFR in male mice (Hackbarth & Hackbarth, 1981; Messow et al., 1980), the present study showed greater glomerular filtration function in female mice who had sex differences in GFR. This discrepancy was probably due to approach of analysis of GFR. In our study, GFR values were normalized to BW, different from other studies in which GFR was taken as the whole animal (Hackbarth & Hackbarth, 1981; Messow et al., 1980).

Study on sex differences in renal hemodynamics by direct measurement of RBF is limited, probably due to technical difficulty. Using an ultrasound flow probe placed around the renal artery, we directly measured basal RBF in male and female mice. Similar to sexual influence on GFR, the sex differences in RBF were also strain dependent. There was no significant difference in RBF between male and female C57BL6 mice. Similar results were previously reported in C57BLK/6 mice by Schneider et al. (Schneider et al., 2010). However, in 129/Sv and C57BLKS/J mice, females had significantly greater RBF than males. The lower RBF in female mice must be due to higher RVR as dictated by Ohm's law.

In addition to sex, strain is also a factor affecting GFR and RBF. In the present study, the basal GFR in male 129/Sv mice was significantly lower than that in male C57BL6 mice. Using the same approach, Schock‐Kusch et al. also reported that male adult (12–16 weeks) 129/Sv mice had lower GFR compared to the sex‐ and age‐matched C57BL6 mice. Strain differences in RBF have not been reported previously. In the present study, we found that male 129/Sv mice had significantly higher RBF than the other two strains with the same sex. Although the mechanisms underlying the strain differences are not clear, we could assume that a variety of genetic factors are involved. In addition, inter‐strain differences in kidney weight and structure may also contribute to the differences, as reported by an earlier study (Hackbarth & Hackbarth, 1981; Messow et al., 1980).

NO has been shown to play an important role in regulating renal hemodynamics (Ketteler et al., 1994; Layton & Sullivan, 2019). eNOS has been identified within the kidney and has been localized in the glomerulus and within specific tubular segments and renal vasculature (Neugarten et al., 1997; Sullivan et al., 2010). Numerous studies have demonstrated the effects of sex/gender on NO system with female subjects/animals having greater NOS protein levels (Neugarten et al., 1997), enzymatic activity (Sullivan et al., 2010), NO production (Forte et al., 1998), and NO bioavailability (Chen et al., 2017). In the present study, we found that eNOS had distinct effects on the sex differences in GFR and RBF in C57BLKS/J mice. Although deletion of eNOS reduced the basal GFR in both sexes, the pattern of sex differences was the same as that in WT mice. This suggests that eNOS does not play a significant role in the higher GFR in female mice. Our findings are consistent with a recent report that the plasma creatinine levels were comparable between male and female rats treated with NOS inhibitor, N^ω^‐nitro‐L‐arginine methyl ester (L‐NAME) (Ramirez et al., 2020). Different from effects on GFR, eNOS knockout dramatically reduced RBF only in male mice, possibly attributed to an increase in renal vascular tone (indicated by an increase in RVR and MAP). Our findings are consistent with an earlier study in which inhibition of NOS with L‐NAME significantly decreased RBF in male rats but had no effect in female rats (Reckelhoff et al., 1998). It is not known why the deletion of eNOS did not influence RBF in female mice. One explanation is that the renal vasculature of males is more dependent on NO than the renal vasculature in females. The other possibility includes that other isoforms of NOS, such as neuronal NOS (nNOS) and inducible forms of NOS (iNOS) are predominant in female mice, particularly in C57BLKS/J strain. Indeed, iNOS levels are significantly greater in the renal medulla of female rats compared with male rats (Neugarten et al., 1997). nNOS is predominantly localized in several regions of the kidney where they participate in the control of renal hemodynamics (Sullivan et al., 2010). Under normotensive conditions, female rats have greater renal nNOS immunoreactivity (Wang et al., 2006). Nakano and Pollock reported that in female, but not in male rats activation of nNOS led to diuresis and natriuresis (Nakano & Pollock, 2009).

It is well known that there are pronounced sex differences in the development of renal disease with males developing a more severe pathology faster than age‐matched females (Clotet et al., 2016; Institute for Health Metrics and Evaluation, 2020; Layton & Sullivan, 2019; Maric‐Bilkan, 2020; Neugarten et al., 2000; Skrtic et al., 2017). However, the data from the present study suggest that the sex differences in GFR and RBF were not associated with severity of renal injury in eNOS^−/−^ *dbdb* mice, a well‐known mouse model of type II diabetes. It is true that conflicting evidence exists regarding any sex difference in the development and progression of DN (Layton & Sullivan, 2019; Maric‐Bilkan, 2020). Our findings in the present study are consistent with our previous findings in the same mouse model (Ma, Li, et al., 2019). It is not known whether the lack of sexual dimorphism in this mouse model is attributed to the deletion of eNOS. The present study revealed that knockout of eNOS minimized the sex differences in RBF observed in WT C57BLKS/J mice. Comparable RBF levels between male and female eNOS^−/−^ diabetic mice may lead to a similar course of DN in both sexes.

One limitation to this study is that GFR and RBF were measured under completely different conditions. GFR was measured in awake mice and was the whole‐animal clearance (two kidneys) while RBF was measured in anesthetized, instrumented mice and was from one‐kidney. Therefore, the two parameters should be considered and interpreted separately, and that findings made under one set of conditions should not be projected to the other. For instance, in the present study, female 129/Sv and C57BLKS/J mice had greater GFR, but lower RBF. For the same reason, it is impossible to calculate filtration fraction and estimate the ratio of the afferent arteriolar tone to the efferent arteriolar tone, which are also factors indicating glomerular filtration function.

In summary, the present study showed sex differences in GFR and RBF in mice. The sexual dimorphisms were strain dependent, and might also be age dependent. The sexual influences on GFR and RBF were independent of each other and were mediated by distinct mechanisms. eNOS was not involved in the sex differences in GFR, but contributed to the greater RBF in male mice. Furthermore, the sexual dimorphism did not impact the severity of renal injury in eNOS^−/−^ *dbdb* mice. Since the mouse is the most common animal model used in renal research, our findings suggest that sexual impact should be considered with strain and age of mouse when studying renal function and disease progression.

## AUTHOR CONTRIBUTIONS

Rong Ma and Keisa W. Mathis conceived and designed the study, analyzed data; prepared most figures, and drafted the manuscript. Yu Tao and Cassandra Young‐Stubbs performed experiments, interpreted the results of experiments, and prepared some figures; Parisa Yazdizadeh Shotorbani and Dong‐Ming Su bred and genotyped animals; all authors approved the final version of this manuscript.

## FUNDING INFORMATION

The work was supported by National Institutes of Health Grant R01s (NIH/NIDDK, DK115424‐01 to R. Ma; NIH/NIHL, 1R01HL153703‐01A1 to KW. Mathis), the Translational Project Award from American Heart Association (20TPA35500045, to R. Ma), The Ideal Award from Department of Defense (LR210096 to KW. Mathis), and American Heart Association Predoctoral Fellowship (22PRE903925, to Y. Tao).

## CONFLICT OF INTEREST STATEMENT

All authors declared no competing interests.

## ETHICAL APPROVAL

All animal procedures were approved and performed in accordance with the guidelines and regulations of the Institutional Animal Care and Use Committee of the University of North Texas Health Science Center (UNTHSC). All animals were maintained at the animal facility of UNTHSC under local and National Institutes of Health guidelines.
